# Genome-Wide Identification and Expression Analysis of the *SUC* Gene Family in Peanut (*Arachis hypogaea* L.) Reveals Its Role in Seed Sucrose Accumulation

**DOI:** 10.3390/cimb48010029

**Published:** 2025-12-25

**Authors:** Zongqin Feng, Qinqin He, Yixiong Zheng, Yu Zhang, Xiaolin Chen, Jiping Liu, Xinmin Huang

**Affiliations:** 1 College of Agriculture and Biology, Zhongkai University of Agriculture and Engineering, Guangzhou 510225, China; zongqinfeng@163.com (Z.F.); gdsscqs@163.com (Y.Z.); yzhang0327@163.com (Y.Z.); 2 Guangdong Provincial Key Laboratory for Green Agricultural Production and Intelligent Equipment, College of Biology and Food Engineering, Guangdong University of Petrochemical Technology, Maoming 525000, China; xiaohe1507@126.com (Q.H.); chenxl_rainbow@126.com (X.C.)

**Keywords:** peanut, *SUC* gene family, bioinformatics analysis, sucrose accumulation, expression profile

## Abstract

Sucrose is a key quality trait in peanuts, yet high-sucrose varieties are scarce. Although sucrose transporters (SUT/SUC) play crucial roles in sucrose transport and accumulation during seed development, systematic analyses in peanuts are limited. This study conducted a genome-wide analysis of the *SUC* gene family in cultivated peanut (*Arachis hypogaea* L.). Sixteen *AhSUC* genes were identified and characterized for genomic distribution, phylogeny, and expression across tissues and developmental stages. The genes are unevenly distributed across the genome with clustered chromosomal localization. All AhSUC proteins contain the conserved sucrose/proton co-transporter domain (IPR005989), exhibit the typical 12 transmembrane α-helical structure of the major facilitator superfamily, are hydrophobic, and predicted to localize to the membrane. Promoter analysis revealed cis-regulatory elements associated with growth, development, light, hormone, and stress responses. Expression profiling showed tissue-specific patterns, with eight *AhSUC* genes being highly expressed in cotyledons and embryos. Comparative analysis between high-sugar and conventional varieties showed higher expression of *AhSUC2*, *AhSUC9*, and *AhSUC11* in the high-sugar variety, correlating with increased sucrose accumulation. Functional validation using a sucrose transport-deficient yeast mutant confirmed the sucrose transport activity of these genes. These findings provide insight into sucrose accumulation mechanisms and offer genetic targets for breeding high-sugar peanut varieties.

## 1. Introduction

The cultivated peanut (*Arachis hypogaea* L.), commonly known as the “longevity nut” and also referred to as groundnut or earthnut, is an allopolyploid legume crop. It originated in the Andes Mountains of South America and can be traced back to the wild diploid ancestors, *Arachis duranensis* (AA genome) and *Arachis ipaensis* (BB genome), and was formed through hybridization and domestication [1,2,3]. Not only is it rich in protein resources [4], but it is also a major oilseed crop [5]. Based on their morphology and growth habits, groundnuts (*A. hypogaea* L.) are classified into two subspecies, *A. hypogaea* ssp. *hypogaea* and *A. hypogaea* ssp. *fastigiata*, and six varieties [1,3,4]. Peanuts cultivated worldwide today are primarily oil crops, and peanut oil is highly valued for its higher smoke point and milder flavor compared with those of other vegetable oils [6]. Peanuts have garnered significant attention because of their bioactive components that exhibit potential for cancer prevention and anti-inflammatory effects, showing promise for disease prevention and treatment [5]. As of 2023, China’s annual peanut planting area was approximately 4.83 million hectares, accounting for 25% of the global total, with a total output of approximately 19 million tons, representing 35% of the global peanut production [7].

Recently, fresh peanuts have gained widespread popularity in the consumer market. Peanuts can be consumed directly, preserving nutrients to the greatest extent possible, and are characterized by their sweet and crisp texture, aromatic and non-greasy taste, and easy digestibility. Carbohydrates in peanut kernels are a crucial component of dry matter, playing a vital role in determining flavor quality and directly affecting the quality of deep-processed products, such as candies and baked goods containing peanuts [8,9]. Sucrose is the primary soluble carbohydrate in peanut kernels. Pattee et al. found that the sucrose content in 52 peanut varieties ranged between 20.32 and 42.28 mg/g [8]. Among the 185 popular peanut varieties in China, the lowest sucrose content was only 10.20 mg/g, whereas the highest was 84.80 mg/g [9]. Currently, high-sucrose-content peanut varieties are relatively few in China, because of which meeting the market demand has become challenging. Therefore, breeding of and research on high-sucrose and sweet-tasting edible peanut varieties have garnered increasing attention, and controlling sucrose accumulation in peanut kernels is a key direction for breeding high-sucrose varieties [10].

Sucrose is the most important and widely distributed disaccharide required for plant growth and development. It acts not only as a product of photosynthesis in higher plants, but also as a primary form of carbohydrate storage and accumulation [11,12]. Plant growth and development depend on nutrient supply, which in turn relies on the dynamic regulation of source-sink relationships. This relationship constitutes a highly complex and finely tuned transport process that ensures that plants maintain normal physiological functions across different growth stages and environmental conditions [13]. Therefore, the synthesis, transport, and utilization of sucrose are tightly regulated, and alterations in these processes can profoundly affect plant growth and development [14,15].

Sucrose transport pathways primarily include the apoplastic and symplastic pathways [16,17]. Long-distance transport and distribution of photosynthetic nutrients from the source to the sink require sugar transporters. Three key types of sugar transporters exist in most plants: sucrose transporters (SUT/SUC), sugar-exported transporters (SWEET), and monosaccharide transporters (MST) [18]. Among these, SUCs are a class of membrane-bound proteins with sucrose transport capability and are widely distributed in tissues and cells of higher plants. They actively load sucrose onto the sieve element-companion cell complex in phloem sieve tubes, making SUCs crucial for photosynthetic carbon allocation between source and sink tissues [19,20]. Typically containing 12 transmembrane (TM) α-helices, a characteristic of major facilitator superfamily (MFS) proteins, SUCs are widely distributed throughout plants. Phylogenetic analyses indicated that angiosperm SUCs form three major clades, designated by Aoki et al. as types I, II, and III [21]. Type II SUCs are subdivided into ancestral forms, specifically Type IIA SUCs, which are present in all land plants. Type IIB SUCs are unique to monocotyledons and include phloem-loading transporters in these species [22].

Most SUTs function as energy-dependent proton-coupled sucrose/H^+^ symporters, utilizing proton motive force to transport sucrose across membranes against concentration gradients. *Arabidopsis thaliana*’s *AtSUC1* represents one of the earliest identified H^+^-coupled sucrose transporters in plants [23]. Spinach *SoSUC1* was the first SUC to be isolated [24]. With the gradual release of plant genomic data and rapid development of molecular biology, breakthroughs have been made in elucidating the functions of SUCs in various plants. The functions and regulatory mechanisms of SUCs have been reported in numerous model plants, including *Arabidopsis* [25,26,27], maize [28,29], sorghum [30,31], and rice [32,33]. Additionally, functions of SUCs have been studied in some fruits, including strawberries [34], apples [35], and peaches [36]. These studies have laid the foundation for studying the *SUC* gene family while also providing insights into the molecular mechanisms of sugar accumulation in plants.

Considering that SUC plays a crucial role in regulating sugar accumulation in fruits, studying the function of SUC in sugar accumulation in peanut kernels is important for breeding high-sucrose peanut varieties. However, current reports on peanut *SUC* genes remain limited, particularly with respect to genome-wide identification of the peanut *SUC* gene family and their roles in sucrose accumulation in peanut seeds. To address this, we conducted genome-wide identification of the *SUC* gene family in cultivated peanut (*A. hypogaea*) by analyzing gene structure, conserved motifs, phylogenetic relationships, synteny, promoter cis-regulatory elements, and tissue expression patterns. In addition, we investigated the expression characteristics of *SUC* genes and their sucrose transport functions in high-sugar peanut varieties. The objective of this study was to systematically characterize the *SUC* gene family in peanut at the whole-genome level and to elucidate their roles in sucrose accumulation in seeds. Our findings will provide a theoretical foundation and technical support for understanding the molecular mechanisms of sugar transport and accumulation in peanut, which will be useful for breeding high-sugar varieties. This advancement will promote diversification of the peanut industry from traditional oilseed production to fresh consumption, processing, and other applications, thereby enhancing economic benefits and market competitiveness.

## 2. Materials and Methods

### 2.1. Identification of SUC Genes in Peanut

The genome of *A. hypogaea* cv. Shitouqi was obtained from the Peanut Genome Resource (PGR) database (https://pgr.itps.ncku.edu.tw/download_genomic_data.php, accessed on 18 July 2025) [37]. Use nine *Arabidopsis thaliana* AtSUC proteins to align with the peanut genome and screen candidate AhSUC protein sequences. Candidate proteins containing sucrose/proton co-transporter (GPH_sucrose, IPR005989) conserved domains were then identified using an NCBI Batch CD Search (https://www.ncbi.nlm.nih.gov/Structure/bwrpsb/bwrpsb.cgi, accessed 20 July 2024). Additionally, TM structures were predicted using TMHMM-2.0 (https://services.healthtech.dtu.dk/services/TMHMM-2.0/, accessed 20 July 2025) to screen for members containing the typical 12 TM α-helical structure of the MFS proteins [38].

### 2.2. Chromosomal Localization and Physicochemical Properties

Based on annotated peanut genome data, TBtools (version 2.376) [39] was used to perform chromosomal physical mapping and visualization of *AhSUC* gene family members. *AhSUC* genes were named according to their chromosomal order. Protein molecular weight and isoelectric point predictions for peanut *AhSUC* candidate genes were performed using ExPASy (version 3.0) (https://web.expasy.org/protparam/, accessed on 25 July 2025). Subcellular localization was predicted using ProtComp (version 9.0) (http://www.softberry.com/berry.phtml?topic=protcomppl&group=programs&subgroup=proloc, accessed on 25 July 2025).

### 2.3. Phylogenetic Analysis of the SUC Gene Family

Multiple alignments of SUC amino acid sequences from peanut, *Arabidopsis*, and rice were performed using the MEGA11 (version 11) [40]. The alignment results were used to construct a phylogenetic tree using the neighbor-joining (NJ) method with 1000 bootstrap replicates. The tree was visualized using the iTOL online tool (https://itol.embl.de/, accessed on 21 August 2025).

### 2.4. Gene Structure and Analysis of Conserved Motifs

Conserved motif analysis of the AhSUC protein sequence was performed using the online tool, MEME Suite 5.5.8 (https://meme-suite.org/meme/, accessed on 21 August 2025) [41]. The maximum number of motifs was set to 10, and all other parameters were set to their default values. Based on the MEME results, motif patterns were redrawn using TBtools (version 2.376) [39]. An NJ phylogenetic tree was constructed using the MEGA11 (version 11). Genome annotation files were analyzed for gene structure using TBtools [39], integrating phylogenetic analysis of all AhSUCs and conserved motif analysis for a more detailed representation. Batch CD Search analysis was performed on 16 target sequences, and TBtools [39] was used to visualize conserved structural domains.

### 2.5. Protein Structure Analysis of Peanut AhSUCs

To gain more insights into the structural characteristics of AhSUCs in peanut, the online tool SOPMA (https://npsa-prabi.ibcp.fr/, accessed on 23 August 2025) was used to predict and analyze their secondary structures. Following secondary structure analysis, SWISS-MODEL (https://swissmodel.expasy.org/, accessed on 23 August 2025) was used for tertiary structure modeling.

### 2.6. Promoter Cis-Element Analysis

The upstream sequence (2 kb) of the peanut *AhSUC* coding region was retrieved from the genome sequence using TBtools (version 2.376) [39]. This sequence was then submitted to PlantCARE [42] (https://bioinformatics.psb.ugent.be/webtools/plantcare/html/, accessed on 24 August 2025) to identify regulatory elements. The data obtained were visualized using TBtools (version 2.376) [39].

### 2.7. Collinearity Analysis

Genome and annotation information for soybean and *A. thaliana* were downloaded from the NCBI database. Based on the genomic annotation of the peanut *AhSUC* gene, collinearity analysis with *Arabidopsis* and soybean was performed using the multiple collinearity scanning tool (MCScanX) in TBtools (version 2.376) [39]. The collinearity relationships of *AhSUC* were visualized using the advanced Circos tool in TBtools (version 2.376) [39]. The Ka/Ks values for homologous gene pairs of peanut *AhSUCs* were calculated and analyzed using the simple Ka/Ks Calculator tool in TBtools (version 2.376) [39].

### 2.8. Gene Expression Analysis

Based on the gene IDs of the peanut *SUC* gene family, expression levels in different tissues and developmental stages of the peanut cultivar Shitouqi were retrieved from the PGR database (https://pgr.itps.ncku.edu.tw/transcriptome.php, accessed on 30 July 2025). The analyzed tissues included cotyledon, embryo-I, embryo-II, embryo-III, embryo-IV, florescence, gynophore, leaf, pericarp-I, pericarp-II, pericarp-III, root, root and stem, root nodule, root tip, stem, stem tip, testa-I, and testa-II.

### 2.9. Plant Materials and Determination of Sucrose Content

In this study, two peanut varieties with contrasting sugar contents were used: Zhongkaihua 1 (low-sugar variety, ZKH) and Nanbeihong (high-sugar variety, NBH), both bred by the Zhongkai Agricultural Engineering College. The plants were cultivated under open-field conditions at the experimental base of the Guangdong University of Petrochemical Technology, with each variety planted in separate plots spaced 20 cm × 30 cm apart. Approximately 100 plants per variety were grown to ensure that biological replicates were available. Sampling was conducted at three critical stages of seed development: early development (20 days after pollination, DAP, S1), seed-filling stage (40 DAP, S2), and physiologically mature stage (60 DAP, S3). Thirty seeds from the same developmental stage were pooled as one biological sample, rapidly frozen in liquid nitrogen, and stored at −80 °C. Three biological replicates were used for each developmental stage.

Approximately 100 mg of the peanut sample was ground in liquid nitrogen, followed by the addition of 2 mL of deionized water. The mixture was extracted at 80 °C for 30 min in a water bath. After cooling, the extract was centrifuged at 10,000× *g* for 10 min, and the supernatant was collected. The sucrose content of the supernatant was determined using the resorcinol spectrophotometric method [43].

### 2.10. RNA Isolation and Quantitative Reverse Transcription Polymerase Chain Reaction (qRT-PCR)

Total RNA was extracted from the three varieties at all three stages using a FastPure Universal plant total RNA isolation kit (Vazyme, Nanjing, China). Reverse transcription was performed following the protocol of the HiScript^®^ II Q RT SuperMix for qPCR (+gDNA wiper) kit (Vazyme). Using cDNA synthesized from the reverse transcription as the template and *AhActin* as the internal reference gene, qRT-PCR analysis was performed on the peanut *AhSUC* gene. The primer sequences are shown in Table S1 (primers were synthesized by Guangzhou Tianyi Huiyuan Gene Technology Co., Ltd. (Guangzhou, China)).

The qRT-PCR system of 20.0 μL consisted of 10.0 μL of 2× ChamQ Universal SYBR qPCR master mix (Vazyme), 0.4 μL each of the forward primer and reverse primer, 2.0 μL cDNA, and 7.2 μL ddH_2_O. The PCR program was as follows: 95 °C pre-denaturation for 30 s; 95 °C denaturation for 10 s, 60 °C extension for 30 s, repeated for 40 cycles. Relative gene expression was calculated using the 2^−ΔΔCt^ method [44].

### 2.11. Verification of Sucrose Transport Capacity of Peanut AhSUC

The sucrose transport capacity of peanut *AhSUCs* was verified by heterologous expression in the sucrose transport-deficient yeast strain, SUSY7/ura3, using the yeast expression vector, pDR196. PCR was performed using peanut cDNA as the template (primer sequences are shown in Table S1). pDR196 was digested using EcoRI and XhoI. The pDR196-AhSUCs recombinant plasmid was constructed using the ClonExpress II One Step cloning kit (Vazyme). The plasmid was transformed into SUSY7/ura3 using the PEG/LiAc method (30 min heat shock at 30 °C, followed by 15 min heat shock at 42 °C), plated on SC/–Ura medium (Coolaber, Beijing, China), and incubated at 29 °C. Single clones were selected and cultured in SC/–Ura broth containing 2% glucose until the optical density at 600 nm reached 0.6–0.8. Serial dilutions of the cultures were prepared, and 1.5 μL of each dilution was dispersed onto both SC/–Ura medium containing 2% sucrose and SC/–Ura medium containing 2% glucose. The plates were incubated at 29 °C for 3–4 days. Colony growth was observed using SUSY7/ura3 cells carrying an empty pDR196 vector as the control.

### 2.12. Statistical Analysis

All experimental data were obtained in triplicate. Data organization, analysis, and chart generation were performed using Microsoft Excel and GraphPad Prism 10.2.3. Duncan’s multiple range test was used to perform multiple comparison analyses of sucrose content and gene expression.

## 3. Results

### 3.1. Identification and Physicochemical Analysis of SUC Genes in Peanut

**Figure 1:**
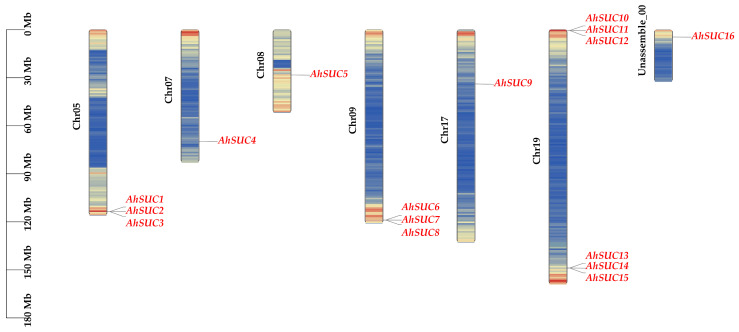
Distribution of *AhSUC* genes on peanut chromosomes. Chr05, Chr07, Chr08, Chr09, Chr17, Chr19, and Unassemble-00 represent the chromosome designations of peanut. The scale on the left indicates chromosome length. Different colors in the figure represent gene density.

Sixteen *AhSUC* genes were identified in the cultivated peanut genome and sequentially named *AhSUC1* to *AhSUC16* based on their chromosomal localization (Figure 1). All 16 AhSUCs encode proteins containing the conserved sucrose/proton co-transporter domain and exhibit 12 TM α-helical structures, consistent with the characteristics of the MFS proteins (Table 1).

The *AhSUC* genes are distributed across chromosomes A05, A07, A08, A09, A17, A19, and Unassemble-00. Among these, chromosome A19 harbored the highest number of *AhSUC* genes (six), followed by chromosomes A05 and A09 (three each), whereas chromosomes A03, A07, A08, A13, A17, and Unassemble-00 each contained only one member. The results indicated that *SUC* genes were widely but unevenly distributed across the peanut genome.

The physicochemical properties of AhSUCs were predicted. The 16 *AhSUC* genes encode proteins ranging in length from 499 to 521 amino acids. The molecular weights of these AhSUC proteins fall between 52,994.20 and 55,019.54, with their theoretical isoelectric points ranging from 7.58 to 9.53. All proteins were classified as basic proteins. The results of predicted subcellular localization indicated that all 16 SUC proteins were possibly localized in the plasma membrane. All AhSUC proteins exhibited positive Grand Average of Hydropathy (GRAVY) values (ranging from 0.494 to 0.596), suggesting that these proteins are hydrophobic (Table 1).

### 3.2. Analysis of AhSUC Protein Motifs and Gene Structure

**Figure 2:**
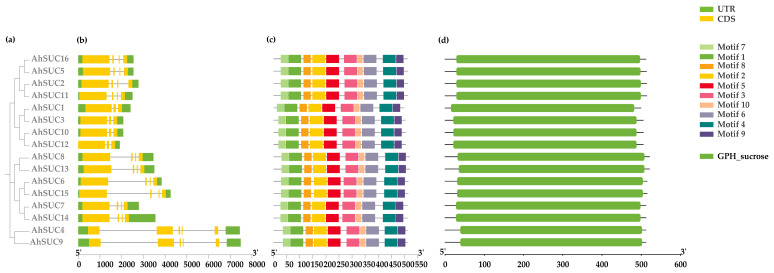
Analysis of AhSUC protein motifs and gene structure in peanut. (**a**) Phylogenetic relationships. (**b**) Gene structure of AhSUC proteins. Exons are shown in yellow, and introns are indicated by black lines. (**c**) Conserved motif analysis. (**d**) Analysis of conserved domains in AhSUC proteins.

To gain deeper insight into the structural diversity of peanut *SUT* genes, we analyzed their exon-intron organization based on genomic sequences. The results revealed significant variation in the number of exons and introns among the three peanut *SUT* genes, ranging from three to five exons and two to five introns (Figure 2a,b). Notably, a substantial proportion of the peanut *SUT* genes contained four exons (10 of 16, 62.5%). Phylogenetic analysis indicated that peanut *SUT* genes within the same clade shared similar gene structures. These findings demonstrated the structural conservation and diversity of peanut *SUC* genes.

Further analysis of the conserved motifs in cultivated peanut AhSUC proteins revealed that all 16 peanut *AhSUC* genes contained identical conserved motifs (Figure 2c). Parallel analysis of conserved domains confirmed that all peanut AhSUCs possessed a sucrose/proton co-transporter (GPH_sucrose, IPR005989) domain (Figure 2d), indicating high domain conservation in peanut SUC proteins.

### 3.3. Phylogenetic Tree Analysis in Peanut

**Figure 3:**
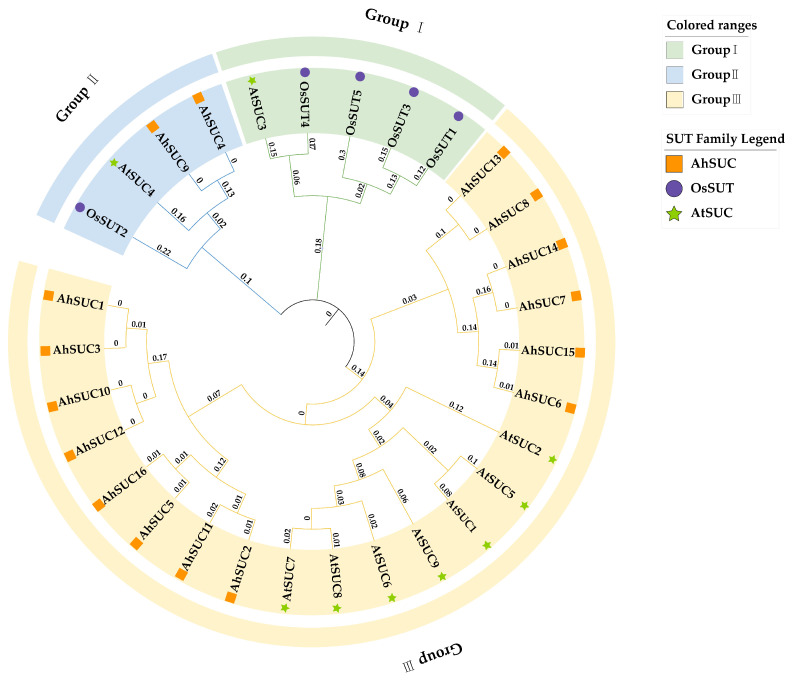
Phylogenetic tree of SUC proteins in *Arachis hypogaea*, *Arabidopsis thaliana*, and *Oryza sativa*. The phylogenetic analysis of SUC-related gene families from the three species was constructed using the neighbor-joining (NJ) method. In the final phylogenetic tree, different colored backgrounds indicate distinct clades, whereas differently colored shapes represent the species included in the analysis: Ah—*Arachis hypogaea* (light green, star); Os—*Oryza sativa* (purple, circle); At—*Arabidopsis thaliana* (orange, square).

Previous studies on *Arabidopsis* and rice have shown that SUTs can be classified into three groups: Group III includes *AtSUC1*, *AtSUC2*, *AtSUC5*, *AtSUC7*, *AtSUC8*, and *AtSUC9*; Group I includes *AtSUC3*, *OsSUT1*, *OsSUT3*, *OsSUT4*, and *OsSUT5*; and *AtSUC4* and *OsSUT2* belong to Group II [22,31]. Phylogenetic analysis based on the amino acid sequences of peanut SUCs (*AhSUCs*) in comparison with those from *Arabidopsis* and rice revealed that most *AhSUC* genes belong to Group III SUTs, with only *AhSUC4* and *AhSUC9* classified as Group II SUTs (Figure 3). No Group I *SUT* genes were identified.

### 3.4. Prediction and Analysis of Peanut AhSUC Protein Structure

**Figure 4:**
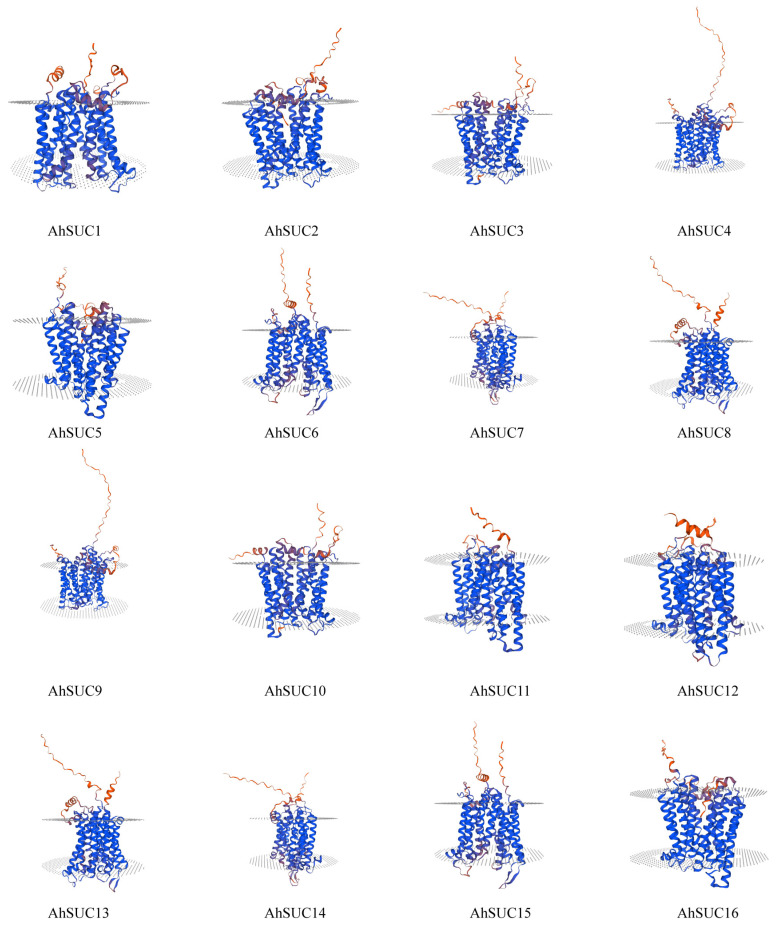
Tertiary structure of the AhSUC proteins.

Next, we analyzed the structure of the peanut AhSUC proteins (Table S2). Analysis of the secondary structure revealed that it primarily consists of α-helices, extended chains, β-turns, and random coils. Among these four secondary structures, the proportion of biological secondary structures in the peanut AhSUC proteins was as follows: α-helices > random coils > extended chains > β-turns. The α-helix content ranged from 42.91% to 47.79%, extended chain content from 13.82% to 17.58%, β-turn content from 2.97% to 4.69%, and disordered coil content from 32.46% to 37.89%. β-Turns constituted the smallest proportion of secondary structures in the AhSUC proteins. The tertiary structures of the AhSUC family proteins were predicted using the SWISS-MODEL website (Figure 4). They were highly similar to the typical 12-TM α-helix proteins. Protein spatial structure is fundamentally determined by amino acid sequence, and each AhSUC protein possesses a distinct sequence with unique features. These sequence-specific variations give rise to differences in structural elements (including α-helices, β-turns, and random coils) among AhSUC proteins, ultimately leading to diversity in their tertiary folding patterns.

### 3.5. Cis-Acting Elements in the AhSUC Gene Promoter

**Figure 5:**
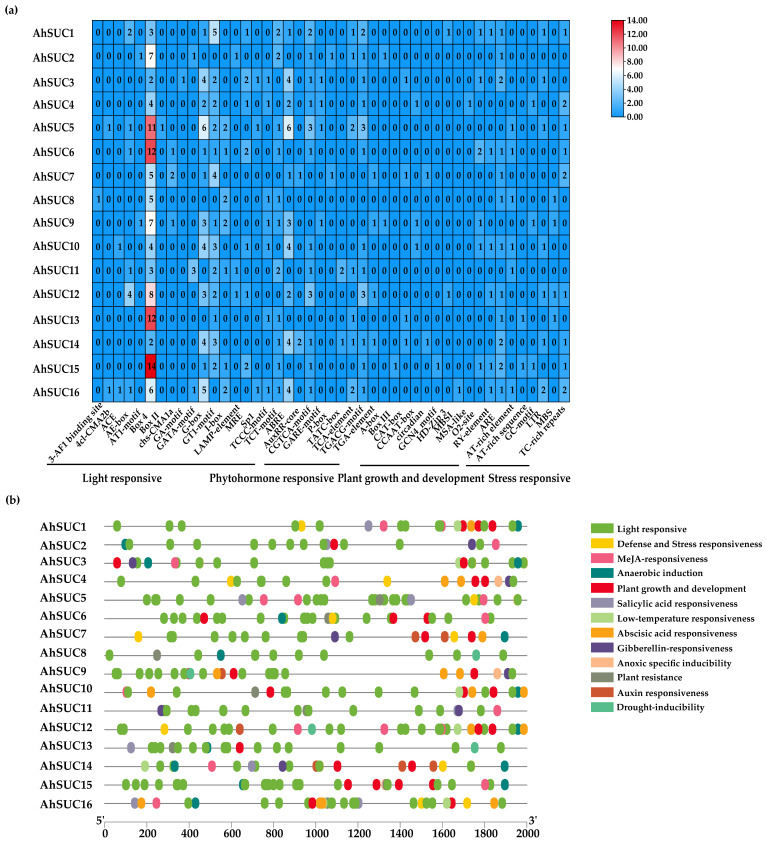
Cis-acting elements of the promoters of *AhSUC* genes. (**a**) Heat map showing the number of cis-acting elements in the putative promoters of *AhSUC*-related genes. Different colors indicate the abundance of cis-acting elements, with red representing the highest number and blue the lowest. (**b**) Distribution of cis-acting elements within the 2000 bp upstream promoter regions of *AhSUC*-related genes. Different types of cis-acting elements are represented by different colors.

Analysis of the 2000 bp upstream sequence of the *AhSUC* gene extracted from the peanut genome database revealed numerous basic cis-acting, light-responsive, hormone-responsive, and stress-responsive elements associated with growth and development (Figure 5a,b). Hormone-responsive cis-acting elements are primarily of five categories: abscisic acid response element (ABRE), gibberellin response element (GARE-motif/P-box/TATC-box), methyl jasmonate-responsive element (CGTCA-motif/TGACG-motif), auxin-responsive element (AuxRR-core/TGA-element), and salicylic acid-responsive element (TCA-element). Among these, ABRE and methyl jasmonate-responsive elements are particularly prevalent and present in over 70% of *AhSUC* gene promoters. Notably, *AhSUC8* lacks hormone-responsive elements (Figure 5a). The cis-acting elements associated with stress responses are primarily of six categories: defense and stress response (TC-rich repeats), anaerobic induction response (ARE), plant resistance response (AT-rich sequence), drought-induced response (MBS), hypoxia-specific induction response (GC motif), and cold response (LTR). These findings suggest that members of the peanut *AhSUC* gene family play crucial roles in regulating seed development and enhancing stress resistance (Figure 5b).

### 3.6. AhSUC Gene Collinearity Analysis

**Figure 6:**
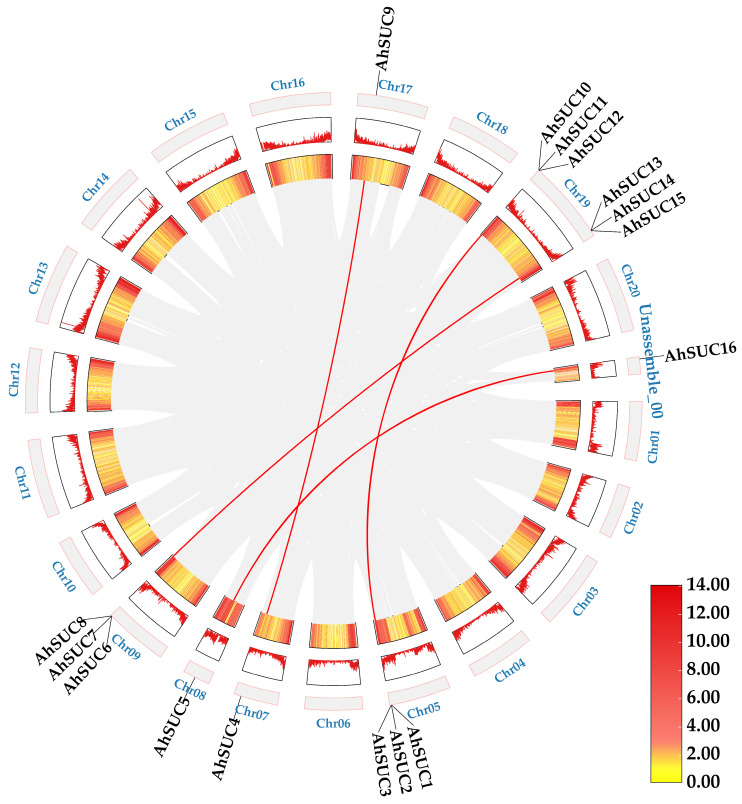
Regional collinearity of *AhSUC* genes in peanut. Red lines indicate duplicated *AhSUC* gene pairs.

Results of the intragenomic collinearity analysis for the peanut *AhSUC* gene (Figure 6) revealed four SUC paralog pairs across the peanut genome, all arising from whole-genome duplication or segmental duplication events. The non-synonymous substitution rate (Ka) for the *AhSUC* gene pairs ranged from 0 to 0.2399 (mean, 0.0666), whereas the Ka/Ks ratio ranged from 0 to 0.1758 (mean, 0.0676) (Table S3). All Ka/Ks means were significantly less than 1 (*p* < 0.01), indicating the occurrence of a strong purifying selection event during evolution and highly conserved function.

**Figure 7:**
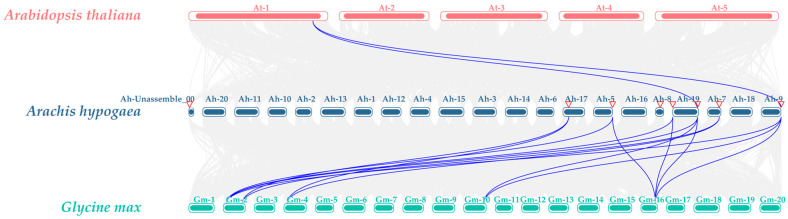
Interspecific collinearity of *SUC* genes among *Arachis hypogaea*, *Arabidopsis thaliana*, and *Glycine max*. Red bands represent *Arabidopsis thaliana* chromosomes, blue bands represent *Arachis hypogaea* chromosomes, and green bands represent *Glycine max* chromosomes. The numbers above the bands indicate chromosome labels. Purple lines indicate collinear gene pairs. The triangles indicate the positions of *AhSUC* on the chromosomes.

Inter-species collinearity analysis of *SUC* between *Arachis hypogaea*, *Arabidopsis thaliana*, and *Glycine max* was performed using TBtools (Figure 7). Only two collinear gene pairs were detected between peanut and *Arabidopsis*, whereas 14 collinear gene pairs were identified between peanut and soybean. These results indicate a higher homology within the *SUC* gene family of peanut and soybean. Therefore, we speculated that these two species may be genetically close or may have undergone similar gene expansion events during the evolution of this gene family. This further indicates the presence of potential common features involved in the regulation of related biological functions.

### 3.7. Analysis of AhSUC Gene Expression Datasets

**Figure 8:**
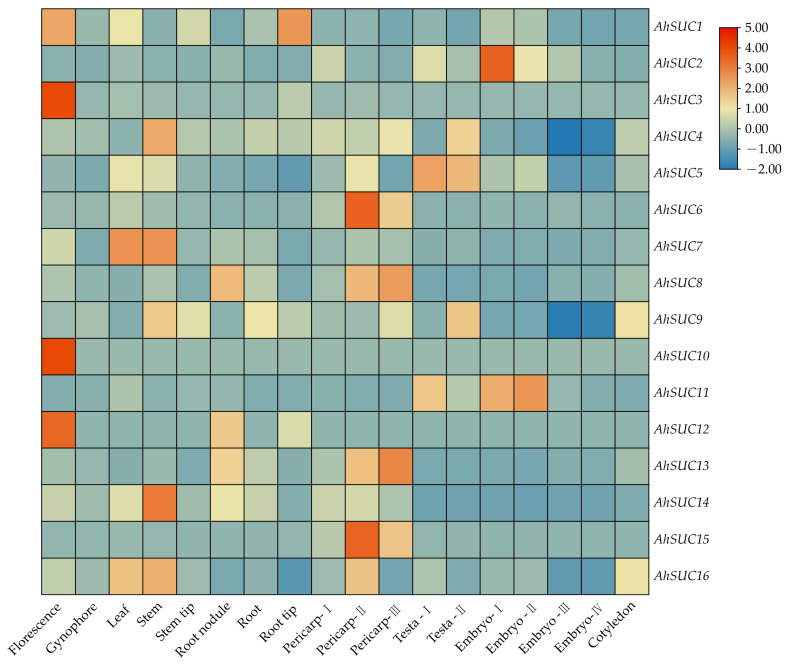
Heatmap of *AhSUC* gene expression across different tissues and developmental stages in the peanut cultivar Shitouqi. The heatmap displays normalized FPKM values.

Analysis of *AhSUC* expression across different tissues and developmental stages of peanut revealed significant tissue-specific differences (Figure 8). *AhSUC1* expression was very low in all tissues and developmental stages. *AhSUC2* was predominantly expressed in the embryo, whereas *AhSUC3* and *AhSUC10* were mainly expressed in flowers, with almost no expression in other tissues. *AhSUC4* was primarily expressed in stems, roots, pericarps, testa, and cotyledons. *AhSUC5* was highly expressed in all tissues at all stages. *AhSUC6* was predominantly expressed in the pericarp, whereas *AhSUC7* was highly expressed in the leaves and stems. *AhSUC8* was highly expressed in root nodules, pericarps, flowers, stems, and cotyledons. *AhSUC9* was highly expressed in stems, roots, pericarps, testa, and cotyledons. *AhSUC11* was mainly expressed in the testa and embryos, whereas *AhSUC12* showed negligible expression across all tissues or stages. *AhSUC13* was highly expressed in the root nodules, roots, pericarps, and cotyledons. *AhSUC14* was highly expressed in the stems, and *AhSUC15* was predominantly expressed in the pericarp. *AhSUC16* was highly expressed in stems, leaves, pericarps, and cotyledons.

### 3.8. Sucrose Content and AhSUC Expression During Peanut Development

**Figure 9:**
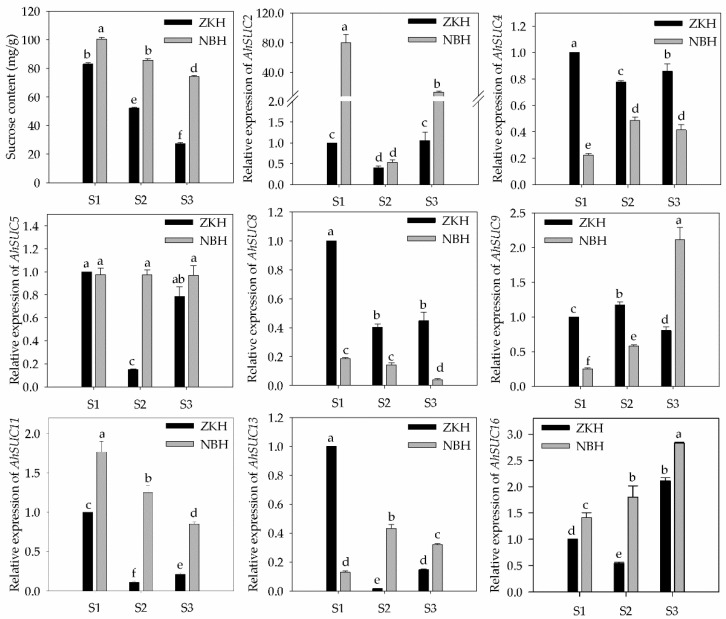
Sucrose content and relative expression of *AhSUC* genes in seeds of two peanut varieties at three developmental stages. ZKH, Zhongkaihua 1 (low-sugar variety); NBH, Nanbeihong (high-sugar variety). S1, 20 days after pollination (early developmental stage); S2, 40 days after pollination (seed-filling stage); S3, 60 days after pollination (physiologically mature stage). Data are presented as the mean ± standard error (*n* = 3). Different letters indicate significant differences (*p* < 0.05) according to Duncan’s multiple range test.

The sucrose content in peanut seeds decreased continuously during development, with Zhongkaihua 1 showing a rapid decline, whereas the high-sugar variety, Nanbeihong, exhibited a slower decrease and maintained significantly higher levels than Zhongkaihua 1 throughout the growth period (Figure 9). We analyzed the expression of eight *AhSUCs* that were highly expressed in the cotyledon and embryo of the two varieties, defining differential gene expression as a relative expression fold change >2, with statistical significance between varieties (Figure 9). *AhSUC2* expression in Nanbeihong was significantly higher than that in Zhongkaihua 1 at S1 and S3, whereas Zhongkaihua 1 showed higher *AhSUC4* expression at all three stages. Difference in *AhSUC5* expression was not observed between the two varieties at S1 and S3, although Nanbeihong showed higher expression in S2. *AhSUC8* expression decreased over time and was consistently higher in Zhongkaihua 1 than in Nanbeihong. *AhSUC9* expression in Zhongkaihua 1 increased initially and then declined, whereas *AhSUC2* expression in Nanbeihong increased and was significantly higher at stage S3. *AhSUC13* expression in Zhongkaihua 1 was significantly higher at S1, whereas that in Nanbeihong was higher at S2, with no difference at S3. *AhSUC16* expression increased over time, with no difference between S1 and S3; however, Nanbeihong showed higher expression in S2. These results showed that the expression of *AhSUC2*, *AhSUC9*, and *AhSUC11* was significantly higher in the high-sugar varieties, which was related to increased sucrose accumulation.

### 3.9. Sugar Transport Capacity of Peanut AhSUC9

**Figure 10:**
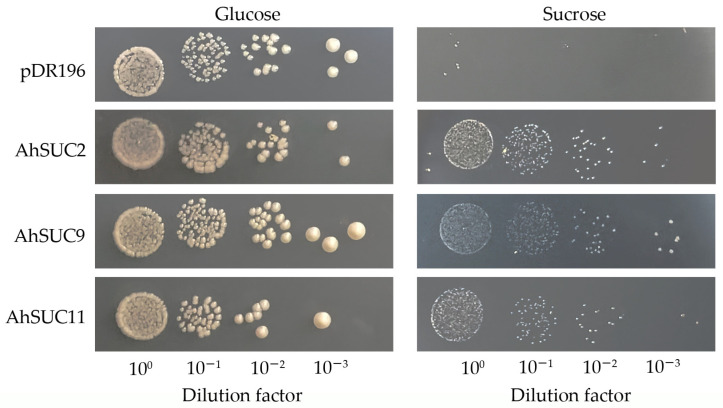
Validation of the functional complementation by *AhSUC2*, *AhSUC9*, and *AhSUC11* in a yeast mutant (SUSY7/ura3). Strains were cultured on SC/–Ura/glucose (**left**) and SC/–Ura/sucrose (**right**) media, with the empty vector (pDR196) used as a negative control. Ten-fold serial dilutions were inoculated onto solid media for incubation and imaging.

The yeast sucrose transport-deficient mutant, SUSY7/ura3, is an invertase-deficient strain that cannot grow on SC-Ura medium with sucrose as the sole carbon source but grows normally on SC-Ura medium supplemented with glucose. To verify the sugar transport activities of *AhSUC2*, *AhSUC9*, and *AhSUC11*, the genes encoding these three proteins were heterologously expressed in SUSY7/ura3. SUSY7/ura3 transformed with the empty vector did not grow on SC/-Ura medium containing 2% sucrose, whereas the yeast transformed with recombinant vectors containing *AhSUC2*, *AhSUC9*, or *AhSUC11* grew normally, indicating that these three proteins possess sucrose transport capabilities (Figure 10).

## 4. Discussion

Sucrose acts not only as a central depot for carbon storage and energy supply but also as a regulator of various physiological processes in plants, such as carbohydrate metabolism, storage protein accumulation, anthocyanin accumulation, and flower induction. In this study, we aimed to provide a comprehensive genome-wide analysis of the *SUC* gene family in cultivated peanut (*A*. *hypogaea* L.), as information on sucrose transport in peanut is limited. SUTs belong to the MFS superfamily of transporters and are characterized by 12 highly hydrophobic TM domains [21,45]. All 16 *AhSUC* genes identified in the cultivated peanut genome contain the 12-TM α-helical structure characteristic of the MFS superfamily. These were concentrated on chromosomes A05, A07, A08, A09, A17, A19, and Unassemble-00, exhibiting a markedly uneven distribution pattern. Notably, chromosome A19 harbored six members, accounting for 37.5% of the total family. Compared to the nine *AtSUC* genes in *Arabidopsis* [26,27], five *OsSUT* genes in rice [13,21,32], and 18 *GhSUT* genes in cotton [46], the number of *AhSUC* genes in peanut is approximately double that in *Arabidopsis*. Expansion of the *SUT* gene family may be a key factor responsible for the accumulation of carbohydrates and lipids in seeds.

Collinearity analysis provided crucial insights into the evolutionary dynamics of the *AhSUC* gene family. The four pairs of paralogs detected within the peanut genome originate from whole-genome or segmental duplications [47]. Moreover, the mean Ka/Ks ratio (0.0676) for all gene pairs was significantly lower than 1, indicating that this gene family underwent strong purifying selection during evolution and exhibits highly conserved functions, consistent with observations in other species [48,49]. Cross-species collinearity analysis revealed 14 *SUC* collinear gene pairs between peanut and soybean, compared to only two pairs with *Arabidopsis*. This suggests that peanut and soybean may share a close genetic relationship in the evolutionary history of the *SUC* gene family or may have undergone similar gene amplification events. We speculated that these three species share common features in the regulation of biological functions related to sucrose transport, laying the foundation for subsequent comparative genomic research.

In recent years, phylogenetic analyses of *SUC* genes have been conducted across numerous species, and a common classification method divides plant SUCs into three clades (Groups I–III) [49,50,51,52,53,54,55]. Group I can be further subdivided into two subgroups: Group IA consists of *SUT* genes from monocots and dicots, whereas Group IB is a monocot-specific subgroup. SUTs in Group II are found in all terrestrial plants, whereas SUTs in Group III are exclusive to dicots. Most *AhSUCs* in peanut belong to Group III SUTs, whereas *AhSUC4* and *AhSUC9* belong to Group II. The absence of Group I *SUT* genes is speculated to be related to plant-specific gene loss events, thus providing new clues for understanding the evolution of the SUT family in different plants.

Protein structure analysis revealed structural conservation and functional relevance within the *AhSUC* gene family. All AhSUC proteins exhibit a characteristic 12-TM α-helical structure, with six N-terminal and six C-terminal helices connected by cytoplasmic loops to form dual pseudo-symmetry. This structure was highly homologous to the structure of *Arabidopsis* AtSUC proteins [45,49], confirming the evolutionary stability of the protein structures of the MFS superfamily. Motif analysis indicated that all 16 AhSUC proteins contained ten conserved motifs in a consistent sequence and possessed a GPH sucrose conserved domain. These conserved elements form the core structural basis for maintaining the sucrose transport function. Furthermore, gene structure analysis revealed that the number of introns (3–5) closely correlated with the evolutionary subfamily classification: Group III genes predominantly contained 3–4 introns, whereas Group II genes primarily possessed five introns. Variations in intron number may influence gene transcription efficiency or mRNA processing, thereby contributing to functional differentiation among subfamilies.

Analysis of cis-acting elements in the promoter revealed multiple functions of *AhSUC* transcriptional regulation. Five hormone response elements were detected in the peanut sucrose transporters, with the ABRE and methyl jasmonate response element (CGTCA-motif/TGACG-motif) being the most widely distributed. *AhSUC8* is a unique member of this family, owing to the absence of all hormone response elements. Notably, *AhSUC8* may participate in basal sucrose transport via non-hormone-dependent pathways as its expression is not induced by stress. This functional differentiation is responsible for the ability of peanut to cope with complex environmental conditions. Hormone- and stress-responsive cis-acting elements form the core molecular basis determining the involvement of this gene in the regulation of stress resistance. For example, abscisic acid and gibberellin can induce *MdSUT1* expression and enhance plant tolerance to abiotic stress [56]. The sucrose transporter, *MdSUT2.2*, promotes sugar accumulation and improves drought tolerance in apple [57]. The pineapple *AcCBF1* directly binds to the CRT/DRE element in the *AcSUT1B* promoter and activates its expression, thereby enhancing cold tolerance [58]. Thus, cis-acting elements of *AhSUC* constitute a crucial defense system for peanut responses to abiotic stresses, providing key theoretical support for stress-resistance mechanisms and the development of stress-tolerant cultivars.

The tissue-specific expression of *SUC* genes confers distinct physiological functions. Transcriptome analysis revealed differential expression of *AhSUC* genes across various tissues, with *AhSUC2*, *AhSUC4*, *AhSUC5*, *AhSUC8*, *AhSUC9*, *AhSUC11*, *AhSUC13*, and *AhSUC16* highly expressed in cotyledons and embryos. Phylogenetic tree analysis indicated that rice *OsSUT4* and *Arabidopsis AtSUC2* are closely related to *AhSUC4* and *AhSUC9*, suggesting that they may perform conserved functions. *OsSUT4* is predominantly expressed in rice mesophyll cells, primary lateral roots, pedicels of fertilized spikelets, seed coat transcellular layers, embryos, and aleurone layers; knockout of *OsSUT4* reduces tiller number and yield in mutant rice lines [59,60]. *DfSUT4* in *Dendrocalamus farinosus* is highly expressed in stems, and its overexpression in tobacco promotes plant growth, with transgenic plants exhibiting taller stems and larger leaves, flowers, and fruits [50]. *Arabidopsis AtSUC5*, together with *AhSUC2*, *AhSUC5*, *AhSUC8*, *AhSUC11*, *AhSUC13*, and *AhSUC16*, belongs to Group III. *AtSUC5* is specifically expressed in the *Arabidopsis* endosperm and participates in the regulation of early seed development [61]. These findings suggest that the genes upregulated in peanut seeds may be involved in sugar accumulation and lipid synthesis. To further clarify the roles of these eight genes in sugar accumulation in peanut seeds, sucrose content and gene expression were compared between high-sugar and low-sugar varieties during fruit development. Sucrose content decreased during seed development in both varieties; however, the high-sugar variety maintained significantly higher sucrose levels than the conventional variety at all three stages. The expression levels of the eight genes also differed between the two peanut varieties during seed development. Among them, *AhSUC2* expression in the high-sugar variety was higher than that in the low-sugar variety at stages S1 and S3. *AhSUC11* exhibited higher expression in the high-sugar variety than in the low-sugar variety across all three developmental stages. Interestingly, although they are homologous genes, *AhSUC4* and *AhSUC9* showed distinct expression patterns between the two varieties: *AhSUC4* expression was higher in the low-sugar variety, whereas *AhSUC9* expression in the high-sugar variety was significantly higher than that in the low-sugar variety at stage S3. This differential expression between varieties may contribute to higher sucrose accumulation in the high-sugar variety, likely resulting from differences in promoter elements between *AhSUC4* and *AhSUC9*, which in turn lead to expression divergence. Functional complementation assays in the sucrose transport-deficient yeast mutant, SUSY7/ura3, demonstrated that all three proteins (*AhSUC2*, *AhSUC9*, and *AhSUC11*) possessed sucrose transport activity. These results indicate that *AhSUC2*, *AhSUC9*, and *AhSUC11* play key roles in sugar accumulation in the seeds of high-sugar peanut varieties. This study reveals the potential roles of *AhSUC2*, *AhSUC9*, and *AhSUC11* in sucrose accumulation in peanut seeds, offering novel targets for breeding high-sugar varieties. However, this study has some limitations. First, the tissue-specific expression of these genes in the two varieties, particularly their expression patterns in different parts of the seed, requires further investigation. In addition, functional validation through overexpression or knockout experiments is needed, and the regulatory mechanisms of these genes remain unclear, especially in naturally high-sugar varieties. Second, the regulatory network of upstream transcription factors has not been systematically elucidated, hindering a comprehensive understanding of their expression regulation. Future research could explore the feasibility of enhancing seed sugar content by increasing the expression of *AhSUC2*, *AhSUC9*, and *AhSUC11* using gene-editing technologies, thereby providing theoretical foundations for the selection of high-quality peanut varieties.

## 5. Conclusions

Using systematic analysis of the peanut sucrose transporter (*AhSUC*) gene family, we identified 16 *AhSUC* genes. These genes exhibited clustered chromosomal distribution patterns, with their expansion primarily attributed to whole-genome or segmental duplication events, showing high homology with soybean *SUC* gene families. Promoter cis-element analysis revealed that the *AhSUC* genes contained various hormone-responsive elements (mainly ABRE and CGTCA-motif/TGACG-motif) and abiotic stress-responsive elements, suggesting their potential involvement in peanut abiotic stress defense systems. Eight *AhSUC* genes were highly expressed in peanut seed embryos and cotyledons. Further analysis indicated that high-sugar peanut varieties had significantly higher sucrose content in seeds than conventional varieties, with *AhSUC2*, *AhSUC9*, and *AhSUC11* showing higher expression levels and sucrose transport activity in the high-sugar varieties. These findings suggest a potential role for these three genes in sucrose accumulation during peanut seed development. Our results have enhanced our understanding regarding the *SUC* gene family in peanut and offered theoretical insights into the mechanisms of sucrose transport, stress resistance, and breeding of high-sugar peanut varieties, thereby providing a foundation for future functional research regarding the role of these genes in sucrose accumulation in seeds.

## Figures and Tables

**Table 1:** Physicochemical properties of *AhSUC* genes.

Name	Cotton Identifier	Chromosome	Protein (aa)	PI	Molecular Weight (Da)	GRAVY	Subcellular Localization	No. of TM
*AhSUC1*	AH05G37290.1	chr5	499	8.37	52,994.20	0.596	PM	12
*AhSUC2*	AH05G37300.1	chr5	514	9.24	54,698.10	0.494	PM	12
*AhSUC3*	AH05G37310.1	chr5	505	8.07	53,622.84	0.576	PM	12
*AhSUC4*	AH07G21450.1	chr7	512	9.53	54,806.05	0.525	PM	12
*AhSUC5*	AH08G13850.1	chr8	513	9.11	54,251.44	0.514	PM	12
*AhSUC6*	AH09G33530.1	chr9	515	9.13	54,319.01	0.550	PM	12
*AhSUC7*	AH09G33540.1	chr9	512	8.99	54,441.84	0.580	PM	12
*AhSUC8*	AH09G33550.1	chr9	521	9.07	54,985.48	0.496	PM	12
*AhSUC9*	AH17G15810.1	chr17	512	9.53	54,806.05	0.525	PM	12
*AhSUC10*	AH19G00570.1	chr19	505	7.58	53,636.82	0.580	PM	12
*AhSUC11*	AH19G00580.1	chr19	514	9.08	54,606.95	0.509	PM	12
*AhSUC12*	AH19G00590.1	chr19	505	7.58	53,593.80	0.590	PM	12
*AhSUC13*	AH19G33930.1	chr19	521	9.07	55,019.54	0.494	PM	12
*AhSUC14*	AH19G33940.1	chr19	512	9.08	54,596.05	0.572	PM	12
*AhSUC15*	AH19G33970.1	chr19	515	9.13	54,304.06	0.555	PM	12
*AhSUC16*	AH00G03340.1	Unassemble-00	512	9.12	54,136.45	0.531	PM	12

Note: GRAVY: Grand Average of Hydropathy. PI: Isoelectric point. PM: plasma membrane. NO. TM: Number of transmembrane domains.

## Data Availability

The original contributions presented in this study are included in the article and Supplementary Material. Further inquiries can be directed to the corresponding authors.

## Supplementary Materials

The following supporting information can be downloaded from https://www.mdpi.com/article/10.3390/cimb48010029/s1.

## Abbreviations

The following abbreviations are used in this manuscript:

SUT	Sucrose transporters
SUC	Sucrose/H^+^ co-transporters
SWEET	Sugars Will Eventually Be Exported Transporters
MST	Monosaccharide Transporters
TM	Transmembrane
MFS	Major facilitator superfamily
NJ	Neighbor-joining
MCScanX	Multiple collinearity scanning tool
qRT-PCR	Quantitative Reverse Transcription Polymerase Chain Reaction
GPH_sucrose	Conserved sucrose/proton co-transporter
GRAVY	Grand Average of Hydropathy
PI	Isoelectric Point
NO. of TM	Number of Transmembrane Domains.
ABRE	Abscisic acid response element
